# Dopamine D_2_ receptor and β-arrestin 2 mediate Amyloid-β elevation induced by anti-parkinson’s disease drugs, levodopa and piribedil, in neuronal cells

**DOI:** 10.1371/journal.pone.0173240

**Published:** 2017-03-02

**Authors:** Jing Lu, Xiaohang Li, Qinying Wang, Gang Pei

**Affiliations:** 1 State Key Laboratory of Cell Biology, CAS Center for Excellence in Molecular Cell Science, Shanghai Institute of Biochemistry and Cell Biology, Chinese Academy of Sciences; University of Chinese Academy of Sciences, Shanghai, P. R. China; 2 School of Life Sciences and Technology, Collaborative Innovation Center for Brain Science, Tongji University, Shanghai, P. R. China; Sungkyunkwan University, REPUBLIC OF KOREA

## Abstract

Although levodopa is the first-line medication for the treatment of Parkinson’s disease (PD) showing unsurpassable efficiency, its chronic use causes dyskinesia. Accordingly, dopamine agonists are increasingly employed as monotherapy or in combination with levodopa to reduce the risk of motor complications. It is well recognized that patients with PD often exhibit cognitive deficits. However, clinical and animal studies assessing the effects of dopaminergic medications on cognition are controversial. Amyloid-β (Aβ) is one of the major hallmarks of Alzheimer’s disease (AD), leading to progressive memory loss and cognitive deficit. Interestingly, the abnormal accumulation of Aβ is also detected in PD patients with cognitive deficits. Evidence indicated that levodopa induced a mild increase of Aβ plaque number and size in the brain of AD mouse. However, the underlying mechanism is unclear. Here we present that both levodopa and piribedil enhance the generation of Aβ and the activity of γ-secretase in human neuronal cells and primary neurons isolated from AD mouse. This effect was reduced by either the antagonism or the knockdown of dopamine D_2_ receptor (D_2_R). We further showed that in the cells expressing β-arrestin 2-biased D_2_R mutant, piribedil promoted cellular Aβ production to the extent comparable to the wild-type D_2_R whereas this activity was absent in those with G protein-biased D_2_R mutant. Moreover, the knockdown of β-arrestin 2 attenuated the increases of Aβ generation and γ-secretase activity mediated by levodopa or piribedil. Thus, our study suggests that targeting D_2_R-mediated β-arrestin function may have potential risk in the modulation of Aβ pathology.

## Introduction

Dopamine is the major neurotransmitter released by dopaminergic neurons to control movement, reward, cognition, and emotion in the central nervous system (CNS). The progressive loss of dopaminergic neurons or the disruption in dopamine signaling leads to many neurological disorders including PD which is characterized by severe movement disorder. Despite dyskinesia accompanied with the long-term use, levodopa, the direct precursor of dopamine, is still a first-line medication for PD treatment showing profound efficacy in the improvement of motor abnormalities. D_2_R is a prominent G protein-coupled receptor (GPCR) mediating dopamine signaling. D_2_R agonists including piribedil are therefore used alone or in combination with levodopa to ameliorate the side-effect. Like many other GPCRs, D_2_R modulates biological functions through both G protein-dependent and independent (i.e. β-arrestin-dependent) signaling. Interestingly, multiple *in vivo* studies have showed that D_2_R/β-arrestin 2-mediated signal pathway plays a key role in dopamine/levodopa-modulated locomotion while the overactivation of G protein signaling is associated with dyskinesia [1–4]. Thus, functional selectivity at D_2_R could achieve therapeutic benefit in the treatment of PD.

It is recognized that PD is more than a motor disorder and often accompanied with cognitive deficit. In addition to striatum, D_2_R is also expressed in the hippocampus correlated with spatial learning and cognitive functions. An age-dependent reduction of D_2_R has been correlated with age-related cognitive deficits [5, 6]. Thus, D_2_R is expected to have positive effect on cognition. However clinical studies assessing the effect of dopaminergic therapy on cognitive functions are controversial, showing improvement in some cognitive symptoms but worsen others [7–9]. In animal experiments, one study using aged rat revealed that chronic treatment with levodopa could trigger cognitive dysfunctions [10]. However in another study using AD mouse, levodopa showed protective effect in learning and memory deficits while both the Aβ plaque number and size were increased by around 10% [11]. Whether and how levodopa regulates Aβ in neuronal cells are unclear.

Aβ is one of the major hallmarks of AD, produced by the sequential cleavage of amyloid precursor protein (APP) by β-secretase and γ-secretase complex. Alternatively, APP is cleaved by α-secretase within the Aβ sequence precluding Aβ generation. GPCRs have been reported to regulate secretase activities via G protein or β-arrestins [12–14]. The present study was set up to examine the effect of dopaminergic medications on Aβ generation in neuronal cells and its underlying mechanism. We showed that both levodopa and piribedil promoted the generation of Aβ and increased the activity of γ-secretase in SK-N-SH cells and human neuronal cells differentiated from induced pluripotent stem cells (iPSCs)-derived neural stem cells (NSCs). In primary neuronal cells isolated from APP/PS1 mouse cortex and hippocampus, an increased Aβ generation after levodopa or piribedil treatment was also detected. Mechanism study showed that these effects were mediated by both D_2_R and β-arrestin 2. These evidences suggest that the therapeutic strategy targeting D_2_R/β-arrestin 2-mediated signal pathway may bring some undesired effects.

## Materials and methods

### Ethics statement

All animal experiments were performed according to the National Institutes of Health Guide for the Care and Use of Laboratory Animals. Protocols were approved by the Biological Research Ethics Committee, Shanghai Institutes for biological Sciences, Chinese Academy of Sciences. Animal pain and discomfort were minimized with efforts. APP/PS1 double-transgenic mice (The Jackson Laboratory, USA, stock number 004462) expressing a chimeric mouse/human APPswe and a human PS1 with exon-9 deletion (PS1ΔE9) were maintained and genotyped according to the guidance of Jackson Laboratory.

### Compounds, reagents, and antibodies

Levodopa, bromocriptine, carbidopa, L-685,458, TAPI-1, Y-27632, and Forskolin (FSK) were purchased from Selleck Chemicals (Houston, TX, USA). Piribedil was from Tocris Bioscience (Bristol, UK). β-Secretase Inhibitor IV (BSI IV) was from Calbiochem (Hayward, CA, USA). Recombinant human BDNF, GNDF, IGF-I were from Peprotech (Rocky Hill, NJ, USA). cAMP and L-Ascorbic acid were from Sigma (St Louis, MO, USA). CellTiter-Glo was from Promega (Madison, WI, USA) and Effectene Transfection Reagent was purchased from QIAGEN (Hilden, Germany). Immunoblotting was performed with the following antibodies: anti-PS1 N-terminus (1–65) (PRB-354P, Covance, Davis, CA, USA); anti-APH1aL C-terminus (245–265) (PRB-550P, Covance); anti-NCT (N1660, Sigma); anti-Pen2 (P5622, Sigma); anti-BACE1 N-termimus (AP7774b, Abgent, Suzhou, China); anti-APP-CTF (A8717, Sigma); anti-sAPPα (secreted Amyloid Precursor Protein-α) (11088, IBL, Hokkaido, Japan); anti-HA (H6908, Sigma); anti-actin (A2066, Sigma); anti-D_2_R (sc-9913, Santa Cruz Biotechnology, Santa Cruz, CA, USA). Immunostaining was performed with the following antibodies: anti-nestin (MAB353, Milipore, Darmstadt, Germany); anti-Sox2 (sc-17320, Santa Cruz); anti-Ki67 (556003, BD Transduction Laboratories, San Jose, CA, USA), and anti-Doublecortin (DCX) (sc-8066, Santa Cruz).

### Cell culture, plasmids, and shRNA

SK-N-SH cells and HEK293 cells were purchased from ATCC. HEK293/APPswe cells were transfected, selected with antibiotics (G418, 1 mg/ml), and maintained in lab. All these cell lines were cultured in Modified Eagle’s Medium (MEM) with 10% (w/v) heat-inactivated fetal bovine serum (FBS) in a humidified incubator with 5% CO2/95% air (v/v) at 37°C. Human iPSCs-derived NSCs were provided by IxCell Biotechnology., Ltd and maintained with neural stem cell culturing basal medium containing 1:50 NSC supplement (IxCell, San Diego, CA, USA).

Full-length D_2_R was subcloned into modified pcDNA3 vector in-frame with Flag at the N-terminus to generate Flag-^[WT]^D_2_R. Or it was used as template for site-directed mutagenesis following an overlapping PCR approach to make Flag-^[Gprot]^D_2_R, Flag-^[βarr]^D_2_R, and Flag-^[A3]^D_2_R constructs. For NanoBRET assay, the WT and mutant D_2_Rs were subcloned into pHTC HaloTag CMV-neo vector (Promega) to make ^[WT]^D_2_R-HT, ^[Gprot]^D_2_R-HT, ^[βarr]^D_2_R-HT, and ^[A3]^D_2_R-HT plasmids. β-arrestin 2 was subcloned into pNLF1-C CMV/Hygro vector (Promega) to generate β-arrestin 2-Nluc plasmid. All the constructs were verified by sequencing.

shRNA targeting human DRD2 or ARRB2 was cloned into pLKO.1 vector following the online protocol (addgene, http://www.addgene.org/tools/protocols/plko/). Targeting sequence for human DRD2: 5’-CACTCCTCTTCGGACTCAATA-3’, and for ARRB2: 5’-GCTAAATCACTAGAAGAGAAA-3’.

### ELISA for Aβ

HEK293/APPswe cells, SK-N-SH cells, and induced human neuronal cells were treated with chemicals at the indicated concentrations for 2 h or 24 h. The conditioned medium was then collected and subjected to a sandwich ELISA for the measurement of total Aβ level. The measurement was done according to the manufacturer’s guidelines. ELISA kits for total human Aβ were obtained from ExCell Bio (Shanghai, China).

### *In vitro* measurement of BACE1 and γ-secretase activities

Fluorogenic substrate assays were carried out as previously reported [15, 16]. Briefly, total membrane fractions were extracted from SK-N-SH cells or induced human neuronal cells and re-suspended in reaction buffers (including 10 μM of specific fluorogenic substrate and vehicle or indicated chemicals). After incubation at 37°C for 30 min (BACE1) or 120 min (γ-secretase), fluorescence of the cleaved substrates was measured by SpectraMax M5 spectrometer (Molecular Devices).

### Differentiation of neuronal cells from human NSCs

The differentiation of NSC into neuronal cells was performed according to the previous reports [17, 18] with minor modification. In details, NSC 13A was detached by accutase and resuspended in NSC medium (Neurobasal (Gibco): DMEM/F12 (Gibco) = 1:1, 1*B27 (Gibco), 1*N2 (Gibco) and 10 μM Y-27632). 5e4 13A per well were seeded in 24-well-plates coated by laminine (Sigma). On the second day, medium was changed to Neuron differentiation medium (Neurobasal: DMEM/F12 = 1:1, 1*B27, 1*N2, 100 nM cAMP, 1 μg/ml Ascorbic acid, 10 ng/ml BDNF, 10 ng/ml GDNF and 10 ng/ml IGF-I). Medium was refreshed every other day.

### Cell viability measurement

Chemical-treated SK-N-SH or mouse primary cells were subjected to the CellTiter-Glo Luminescent Cell Viability Assay (Promega) following the manufacturer’s instructions.

### cAMP assay

The intracellular cAMP was measured using GloSensor^™^ cAMP assay following the manufacturer’s instruction with minor modification (Promega). HEK293 cells were seeded in white 96-well plates (Costar) and co-transfected with pGloSensor^™^-22F cAMP plasmid and WT or mutant D_2_R using Effectene Transfection reagent. Before the cAMP assay, the medium was removed and replaced with the fresh medium containing 2% (v/v) of GloSensor^™^ cAMP reagent. After 90 min incubation at 37°C, cells were equilibrated at room temperature (RT) for 15 min and treated with the ligands at indicated concentrations for another 15 min followed by the measurement of luciferase activity.

### NanoBRET assay

HEK293 cells were cultured in 6-well plates and transiently co-transfected with β-arrestin 2-Nluc and ^[WT]^D_2_R-HT, ^[Gprot]^D_2_R-HT, ^[βarr]^D_2_R-HT or ^[A3]^D_2_R-HT. After overnight incubation, cells were plated into white 96-well plates (Costar) and cultured in Opti-MEM (Life Technologies) containing 4% FBS. HaloTag containing samples were labeled with HaloTag 618 ligand (Promega) at 100 nM of final concentration and incubated at 37°C overnight. The Nano-Glo substrate was added into each well following manufacturer’s instruction (Promega). Donor emission (460 nm) and acceptor emission (610 nm) were measured as a basal signal by SpectraMax M5 spectrometer (Molecular Devices). The chemicals were then added as required and the plate was read.

### Immunofluorescence microscopy

The induced human neuronal cells grown on cover-slip were fixed with 4% paraformaldehyde (PFA) in PBS for 20 min. Cells were permeabilized and blocked with PBS/0.2% Triton X-100/1% BSA for 30 min followed by the incubation with indicated primary antibodies for 2 h at RT. After washing with PBS/1% BSA for three times, cells were incubated with cy3-labeled donkey anti-goat IgG or Alexa Fluor 488-labeled donkey anti-mouse secondary antibodies in the dark for 1 h, washed with PBS/1% BSA, stained with DAPI (1 μg/ml, 10 min), and mounted on slides. Images were acquired using LAS SP8 confocal microscope (Leica, Germany) with a 63 ×/1.40 NA oil objective (Leica).

### Reverse transcription and quantitative real-time PCR

Total RNA was extracted with TRI Reagent (T9424; Sigma) according to the manufacturer’s instructions. Random hexamer primer and MMLV Reverse Transcriptase (M5301; Promega) were used for reverse transcription. All gene transcripts were quantified by quantitative real-time PCR performed with 2 × HotStart SYBR Green qPCR Master Mix (ExCell Bio, Shanghai, China) on a Stratagene Mx3000P (Agilent Technologies). The primers used for the detection of mRNA levels of human DRD1-5 and mouse Drd1-5 were listed as below: DRD1 sense: 5’-AGGGACTTCTCTGTTCGTATCC-3’; DRD1 anti-sense: 5’-GGAACCTGATAACGGCAGCA-3’; DRD2 sense: 5’-CTCTTCGGACTCAATAACGCAG-3’; DRD2 anti-sense: 5’-GACGATGGAGGAGTAGACCAC-3’; DRD3 sense: 5’-AGAAGGCAACCCAAATGGTGG-3’; DRD3 anti-sense: 5’-TGTCGTGGCACTGTAAAGCTC-3’; DRD4 sense: 5’-TCTTCGTCTACTCCGAGGTCCA-3’; DRD4 anti-sense: 5’-TGATGGCGCACAGGTTGAAGAT-3’; DRD5 sense: 5’-CCGTGTCAGACCTTTTCGTG-3’; DRD5 anti-sense: 5’-TGCGCTGAGTCATCTTGCG-3’; Drd1a sense: 5’-CACGGCATCCATCCTTAACCT-3’; Drd1a anti-sense: 5’-TGCCTTCGGAGTCATCTTCCT-3’; Drd2 sense: 5’-ACCTGTCCTGGTACGATGATG-3’; Drd2 anti-sense: 5’-GCATGGCATAGTAGTTGTAGTGG-3’; Drd3 sense: 5’-CCAGTTCACTATCAGCATGGC-3’; Drd3 anti-sense: 5’-CCCCTGTTGTGTTGAAACCAA-3’; Drd4 sense: 5’-GGTGTCGGACCCTACTCAG-3’; Drd4 anti-sense: 5’-GGCAGGACTCTCATTGCCTT-3’; Drd5 sense: 5’-TGCTGTCCAATGAGACACCC-3’; Drd5 anti-sense: 5’-GATGGCGTAGGTTCGGTTCAG-3’. Primers for NSC or neuronal markers: nestin sense: 5’-CTGCTACCCTTGAGACACCTG-3’; nestin anti-sense: 5’-GGGCTCTGATCTCTGCATCTAC-3’; Sox2 sense: 5’-CAAGATGCACAACTCGGAGA-3’; Sox2 anti-sense: 5’-CGGGGCCGGTATTTATAATC-3’; DCX sense: 5’-CATCCCCAACACCTCAGAAG; DCX anti-sense: 5’-GGAGGTTCCGTTTGCTGA-3’; MAP2 sense: 5’-GGGCTGACATCCCACCTA-3’; MAP2 anti-sense: 5’-ATTATTCCACGCTTGCTG-3’; SYN1 sense: 5’-CCCCAATCACAAAGAAATGCTC-3’; SYN1 anti-sense: 5’-ATGTCCTGGAAGTCATGCTG-3’. All the primers were synthesized and purified by Shanghai Sunny Biotechnology Co., Ltd.

### Primary neuronal cell culture

The preparation of mouse primary neuronal cells was performed according to the standard protocols [19, 20] with minor modification. Briefly, after dissection of the cortices and hippocampi from APP/PS1 P0 pups, cells were trypsinized, dissociated, and then seeded in 96-well-plates. The neuronal cells were maintained in 1*B27 and 1*Glutamax (Gibco, 35050)-containing Neurobasal medium. The medium was half refreshed every 4 days. And the compound treatment was performed on DIV8.

### Statistical analysis

All data were analyzed by Prism 6.0 (GraphPad Software Inc., San Diego, CA). Concentration-response curves were analysed using a three parameter non-linear regression analysis. Unpaired Student’s t-test (two-tailed) was applied for the comparisons of two datasets. One-way or Two-way analysis of variance (ANOVA) with Bonferroni’s post-test was used where more than two datasets or groups were compared. Statistical significance was accepted at p < 0.05.

## Results

### Levodopa and piribedil were identified to enhance endogenous Aβ generation and γ-secretase activity in SK-N-SH cells

**Fig 1:**
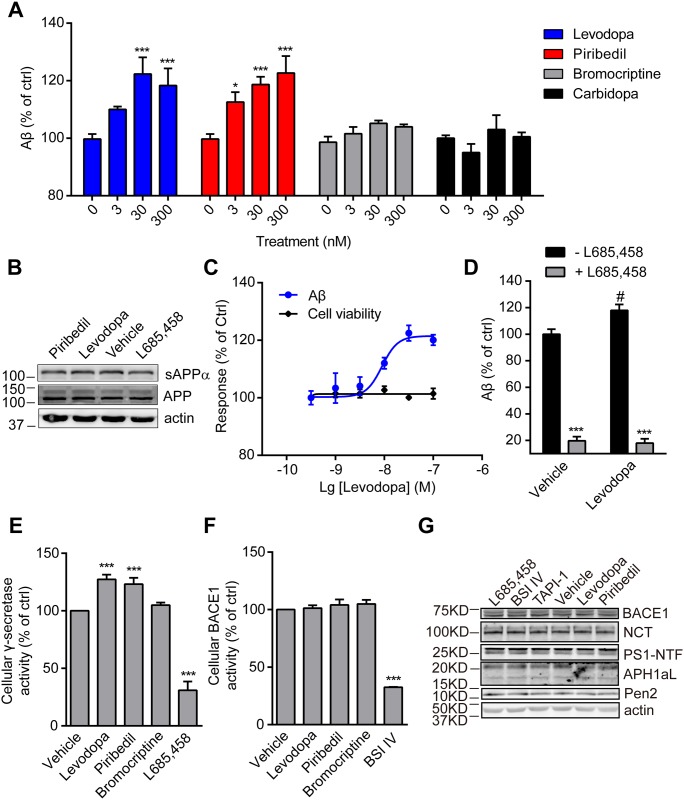
Levodopa and piribedil enhance endogenous Aβ generation and γ-secretase activity in SK-N-SH cells. (A) A focused screening of Aβ generation in response to levodopa, piribedil, bromocriptine or carbidopa at indicated concentrations in SK-N-SH cells. Data are mean + s.e.m., n = 3–4. *p < 0.05; ***p < 0.001 versus the control of each group. (B) Representative image of Western-blot showing the expression of APP and the level of secreted sAPPα in response to the treatments. Actin was used as loading control. (C) The dosage-dependent response curves of Aβ generation and cell viability after the treatment with levodopa at indicated concentrations. Data are mean ± s.e.m., n = 5. (D) The level of Aβ produced by SK-N-SH cells in response to vehicle (control) or levodopa at 30 nM either with or without L685,458 pre-treatment. Data are mean + s.e.m., n = 3–4. #p < 0.05 versus the control within the group; ***p < 0.001 versus the corresponding treatment without L685,458. (E and F) The measurements of γ-secretase (E) and BACE1 (F) activities after the treatment with vehicle (control) or the indicated chemicals. Data are mean + s.e.m., n = 3–5. ***p < 0.001 versus control. (G) Representative image of Western-blot showing the expressions of BACE1 and γ-secretase components (NCT, PS1-NTF, APH1aL, and Pen2) after the treatment with vehicle (control) or indicated chemicals. Actin was used as loading control.

In human neuroblastoma cell line SK-N-SH cells, both levodopa and piribedil significantly promoted endogenous Aβ generation in a dosage-dependent manner whereas carbidopa had little effect (Fig 1A). Bromocriptine, another dopaminergic PD drug, showed a mild tendency to promote Aβ generation with low efficacy. Notably, the expression of APP or the level of soluble APPα (sAPPα) in the culture medium was not affected by either levodopa or piribedil (Fig 1B). Levodopa at micromolar concentrations has been reported to show cytotoxicity in neuronal cells [21, 22]. Here, we showed that levodopa increased Aβ generation with an EC_max_ at 100 nM which has little effect on cell viability (Fig 1C). The effect of levodopa on Aβ generation was effectively prevented by the addition of L685,458, a highly selective inhibitor of γ-secretase (Fig 1D). The increased Aβ production could have resulted from the change of secretase activity. To test this, SK-N-SH cells were challenged with the chemicals followed by membrane preparation and measurement of γ-secretase or BACE1 activity. L685,458 and BSI IV were used as positive controls and significantly inhibited the γ-secretase and BACE1 activity respectively (Fig 1E and 1F). Data showed that stimulation with levodopa or piribedil significantly promoted γ-secretase activity whereas bromocriptine had little effect which is consistent with the results of Aβ generation (Fig 1E). On the other hand, levodopa or piribedil did not change the activity of BACE1 (Fig 1F). Notably, the expressions of γ-secretase components and BACE1 were not altered by the treatments (Fig 1G), suggesting the increased γ-secretase activity is independent of its expression.

### Levodopa and piribedil stimulate Aβ generation and γ-secretase activity through D_2_R

**Fig 2:**
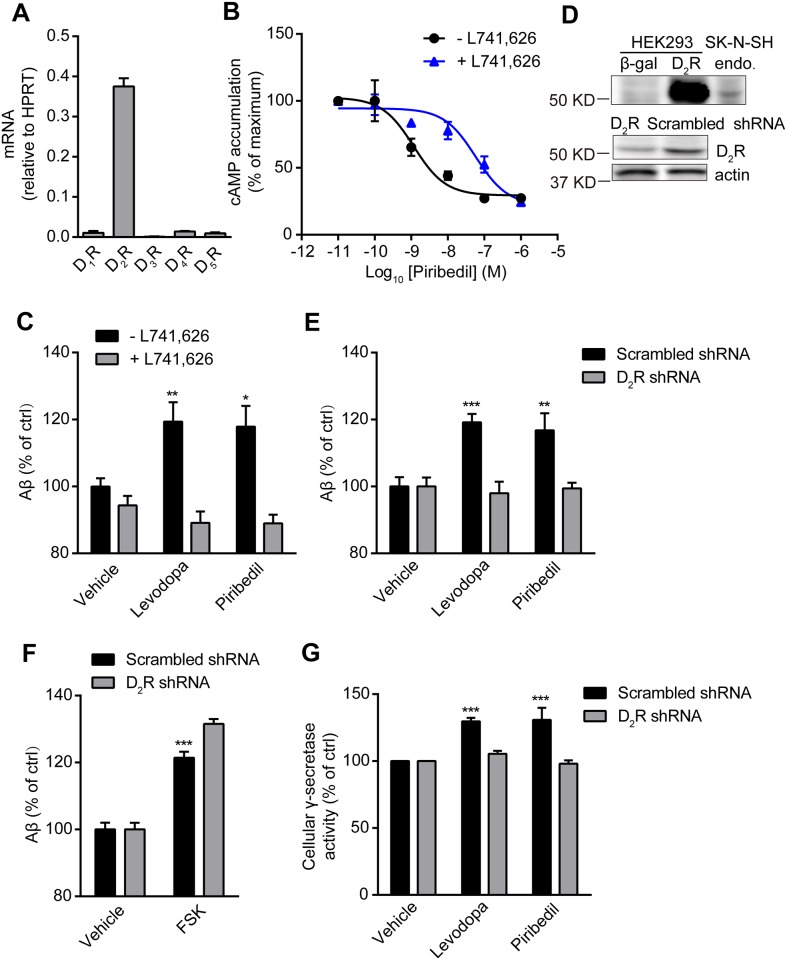
The antagonism or the knockdown of D_2_R reduces levodopa or piribedil-mediated increase of Aβ generation or γ-secretase activity. (A) The mRNA expressions of dopamine receptor subtypes including D_1_R, D_2_R, D_3_R, D_4_R, and D_5_R in SK-N-SH cells. Data are mean + s.e.m., normalized to HPRT. n = 3. (B) The dose-dependent cAMP response after the treatment with piribedil at indicated concentrations in HEK293 cells either with or without L741,626 (1 μM) pre-incubation. Data are mean ± s.e.m., n = 3. (C) Aβ generation in SK-N-SH cells in the absence or presence of 1 μM L741,626. Data are mean + s.e.m., n = 3. *p < 0.05; **p<0.01 versus the control within the group. (D) The protein level of D_2_R in SK-N-SH cells with the infection of scrambled or D_2_R gene specific shRNA. The overexpression of human D_2_R in HEK293 cells indicates that the band at over 50 KD is D_2_R. endo, the endogenous D_2_R in SK-N-SH cells. Actin was used as a loading control. (E and F) Measurement of Aβ level after the stimulation with vehicle (control), levodopa or piribedil at 30 nM (E) or FSK at 1 μM (F) in the cells infected as described in (D). Data are mean + s.e.m., n = 5–6. **p < 0.01; ***p < 0.001 versus the control within the group. FSK, forskolin. (G) Measurement of γ-secretase activity after the stimulation with vehicle (control), levodopa or piribedil at 30 nM in the cells infected as described in (D). Data are mean + s.e.m., n = 3. ***p < 0.001 versus the control within the group.

Levodopa is the precursor of dopamine while piribedil is an agonist of D_2_R and dopamine D_3_ receptor (D_3_R). We examined the mRNA level of dopamine receptors in SK-N-SH cells and found that D_2_R was the most abundant one among all the subtypes (Fig 2A). Therefore, we hypothesized that the effect of levodopa or piribedil on the promotion of Aβ production could be mediated by D_2_R in neuronal cells. To examine this, a selective D_2_R antagonist L741,626 was used to treat the cells before levodopa or piribedil stimulation. Pre-treatment with L741,626 (1 μM) significantly reduced the potency of piribedil-mediated cAMP response indicating the antagonist was effective under this condition (Fig 2B). We further showed that L741,626 (1 μM) numerically but not significantly reduced endogenous Aβ production in SK-N-SH cells while it prevented the elevation of Aβ level induced by levodopa or piribedil (Fig 2C). The endogenous D_2_R expression in SK-N-SH cells was detected at above 50 KD and the infection of gene specific shRNA resulted into an obvious reduction of D_2_R level (Fig 2D). Knockdown of D_2_R did not obviously affect the basal Aβ level but abolished levodopa or piribedil-increased Aβ production (Fig 2E). It did not impair forskolin (FSK)-mediated Aβ response indicating the specificity (Fig 2F). Consistently, levodopa or piribedil-stimulated γ-secretase activity was reduced in the cells with D_2_R knockdown (Fig 2G). All these suggest that levodopa and piribedil increase Aβ generation and γ-secretase activity through D_2_R.

### Levodopa and piribedil stimulate Aβ generation and γ-secretase activity in a β-arrestin 2-dependent manner

**Fig 3:**
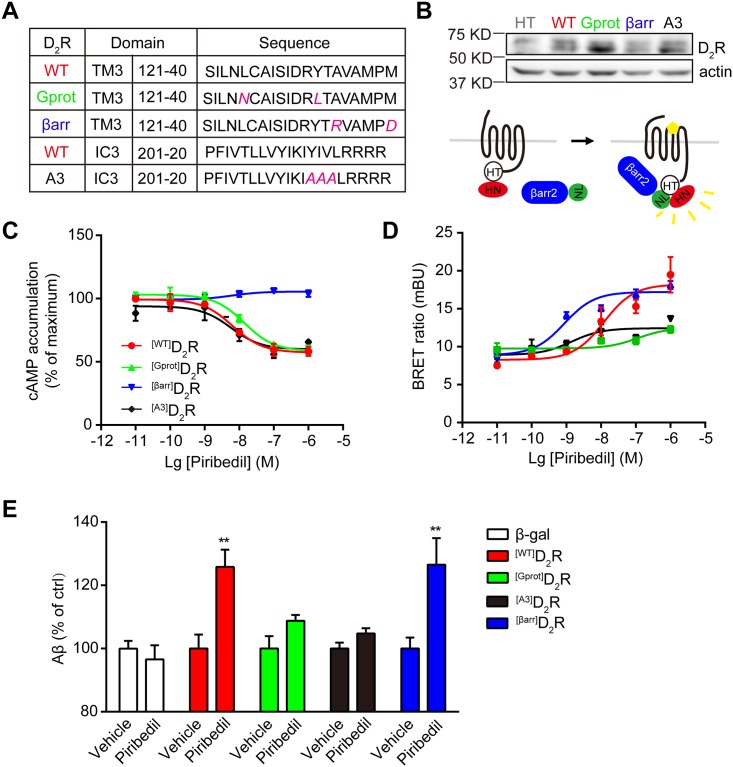
β-arrestin 2-biased D_2_R mediates piribedil-increased Aβ generation. (A) The partial sequence of WT and mutant D_2_R. TM3, the 3^rd^ transmembrane domain; IC3, the 3^rd^ intracellular loop. Gprot, G protein-biased D_2_R; βarr, β-arrestin 2-biased D_2_R; A3, three alanine substitutions at 213–215. The amino acids in red indicate the mutant positions. (B) HEK293 cells were transiently transfected with HT, ^[WT]^D_2_R-HT, ^[Gprot]^D_2_R-HT, ^[βarr]^D_2_R-HT or ^[3A]^D_2_R-HT. After 36 h, the expressions of D_2_Rs were verified. Actin was used as loading control. (C) The cAMP responses after the stimulation with piribedil at indicated concentrations in HEK293 cells transfected with ^[WT]^D_2_R, ^[Gport]^D_2_R, ^[βarr]^D_2_R or ^[A3]^D_2_R. Data are mean ±s.e.m., n = 3. (D) The recruitment of β-arrestin 2-NLuc (βarr2-NL) to the C-terminal HT-tagged ^[WT]^D_2_R, ^[Gport]^D_2_R, ^[βarr]^D_2_R or ^[A3]^D_2_R in response to piribedil at indicated concentrations. NL, Nluc; HT, Halotag. Data are mean ± s.e.m., n = 3. (E) Aβ generation after the treatment with piribedil in HEK293/APPswe cells transfected with ^[WT]^D_2_R, ^[Gport]^D_2_R, ^[βarr]^D_2_R or ^[A3]^D_2_R. Data are mean + s.e.m., n = 4–5. *p < 0.05; **p < 0.01 versus the control within each group.

D_2_R modulates biological functions through both G protein-dependent and β-arrestin-dependent signal pathways. Caron’s lab reported a pair of D_2_R mutations, A135R M140D (^[Gprot]^D_2_R) and L125N Y133L (^[βarr]^D_2_R) within the 3^rd^ transmembrane domain (TM3, Fig 3a), which showed biased signal transduction property for G protein and β-arrestin 2 respectively [4]. Neve’s lab showed that the alanine substitution of residues 213–215 (^[3A]^D_2_R) in the 3^rd^ intracellular loop (IC3, Fig 3a) did not affect G protein activation but impaired β-arrestin 2 recruitment [23]. All these mutants showed little change on cellular expression or distribution compared with the wild-type (WT) receptor (^[WT]^D_2_R) [4, 23].

To distinguish whether piribedil stimulates Aβ production through D_2_R-mediated G protein or β-arrestin signal pathway, we systematically assessed the function of these D_2_R mutations compared with the WT receptor. HEK293 cells have little endogenous expression of dopamine receptors and are easy to be transfected with plasmids. Gs protein-mediated cAMP response and β-arrestin 2 recruitment in response to the treatment were measured using GloSensor^™^ cAMP and NanoBRET assay respectively to confirm the functions of these D_2_Rs. The cells were transfected with relatively low amount of C-terminal HaloTag protein-tagged ^[WT]^D_2_R, ^[Gprot]^D_2_R, ^[βarr]^D_2_R, and ^[3A]^D_2_R as detected by Western-blot (Fig 3B). Piribedil stimulated cAMP response in the cells transiently transfected with ^[WT]^D_2_R-HT, ^[Gprot]^D_2_R-HT, or ^[3A]^D_2_R-HT showing consistence in potency (EC_50_ is around 10 nM). However, little response was observed in those with ^[βarr]^D_2_R-HT (Fig 3C). Few evidences have demonstrated the efficiency of piribedil on β-arrestin 2 recruitment. Here, we used the NanoBRET assay and presented that in the cells expressing ^[WT]^D_2_R-HT, piribedil stimulated β-arrestin 2-Nluc recruitment in a dosage-dependent manner with an EC_50_ at around 10 nM (Fig 3D). It was more potent when ^[βarr]^D_2_R-HT presented but impaired with the expression of ^[Gprot]^D_2_R-HT or ^[3A]^D_2_R-HT. Interestingly, piribedil stimulated Aβ production in the cells expressing ^[WT]^D_2_R or ^[βarr]^D_2_R, which was ameliorated in those with either ^[Gprot]^D_2_R or ^[3A]^D_2_R (Fig 3E). Compared to the D_2_R, the expression of D_1_R in HEK293/APPswe cells did not influence the Aβ generation after treatment with piribedil (S1B Fig). The evoked cAMP accumulation in response to dopamine indicates the successful expression of D_1_R (S1A Fig) whereas piribedil failed to mediate cAMP generation suggesting its poor affinity for D_1_R.

**Fig 4:**
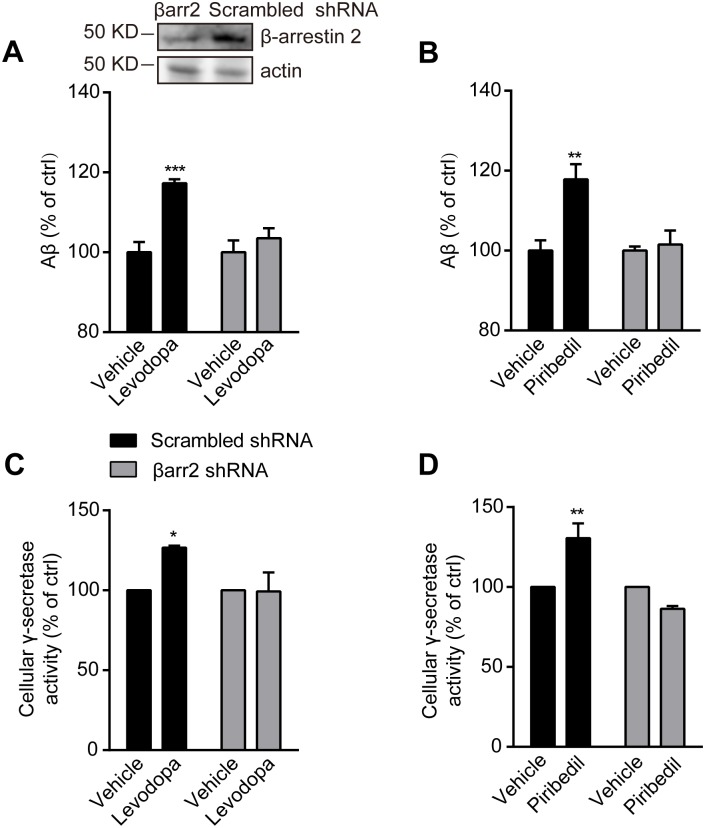
Levodopa or piribedil-mediated increases of Aβ generation and γ-secretase activity are reduced by the knockdown of β-arrestin 2. (A and B) Aβ generation after the challenge with vehicle (control), levodopa (A) or piribedil (B) in SK-N-SH cells infected with scrambled or βarr2 gene specific shRNA. Data are mean + s.e.m., n = 4. **p < 0.01; ***p < 0.001 versus the control within the group. Western-blot image showing the knockdown efficiency. Actin was used as loading control. (C and D) The activity of γ-secretase in response to vehicle (control), levodopa (C) or piribedil (D) in SK-N-SH cells infected with scrambled or βarr2 gene specific shRNA. Data are mean + s.e.m., n = 3. *p < 0.05; **p < 0.01 versus the control within the group.

To confirm the role of β-arrestin 2 in SK-N-SH cells, the gene specific shRNA was introduced. The expression level of β-arrestin 2 was reduced with shRNA expression (Fig 4A). The knockdown of β-arrestin 2 reduced levodopa or piribedil-increased Aβ production (Fig 4A and 4B) and γ-secretase activity (Fig 4C and 4D). Collectively, levodopa and piribedil may mediate Aβ generation through D_2_R-mediated β-arrestin 2-dependent signal pathway.

### Levodopa and piribedil enhance Aβ generation and γ-secretase activity in induced human neuronal cells

**Fig 5:**
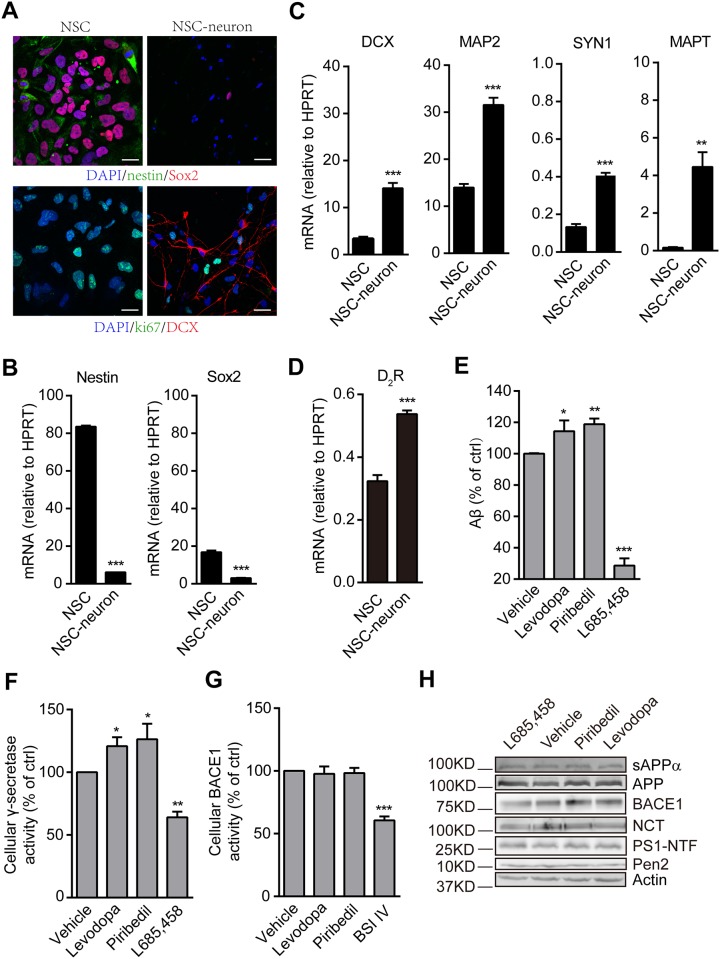
Levodopa and piribedil enhance Aβ generation and γ-secretase activity in induced human neuronal cells. (A) Human NSC and its induced neuronal cells stained with DAPI, nestin, Sox2, ki67 or DCX. Scale bar, 20 μm. (B) qPCR quantification of NSC-expressing genes, nestin and Sox2. Data are mean + s.e.m., n = 3. ***p < 0.001 versus NSC. (C) qPCR quantification of neuron-expressing genes, DCX, MAP2, SYN1, and MAPT. Data are mean + s.e.m., n = 3. **p < 0.01; ***p < 0.001 versus NSC. (D) qPCR quantification of D_2_R in NSC and its induced neuronal cells. Data are mean + s.e.m., n = 3. ***p < 0.001 versus NSC. (E) Aβ generation after the challenge with vehicle (control), levodopa, piribedil or L685,458. Data are mean + s.e.m., n = 6. *p < 0.05; **p < 0.01; ***p < 0.001 versus the control. (F) Measurement of cellular γ-ecretase activity with vehicle (control), levodopa, piribedil, or L685,458 treatment. Data are mean + s.e.m., n = 4. *p < 0.05; **p < 0.01 versus the control. (G) Measurement of cellular BACE1 activity with vehicle (control), levodopa, piribedil, or BSI IV treatment. Data are mean + s.e.m., n = 4. ***p < 0.001 versus the control. (H) Western-blot showing the level of sAPPα in the culture medium and the expression of APP, BACE1, γ-secretase components including NCT, PS1-NTF, and Pen2 in the cell lysate. Actin was used as loading control.

To confirm the effects of levodopa and piribedil in a more relevant system, human neuronal cells were differentiated from iPSC-derived NSCs. The induced neuronal cells were stained positive for DCX, a neuronal marker, while the expressions of nestin and Sox2, two NSC markers, were markedly reduced (Fig 5A). Moreover, the differentiated cells showed low ability in proliferation as indicated by reduced expression of ki67, a proliferation marker. Further, the transcription level of nestin and Sox2 were down-regulated in the induced neuronal cells (Fig 5B) and meanwhile microtubule-associated protein 2 (MAP2), synapsin 1 (SYN1), and microtubule-associated protein Tau (MAPT) were all up-regulated, indicating the formation of neurons in the cell population (Fig 5C). Furthermore, the genes encoding D_2_R was also up-regulated while differentiation (Fig 5D). In these cells, levodopa and piribedil consistently enhanced Aβ generation (Fig 5E) and γ-secretase activity (Fig 5F) while had no effect on BACE1 activity (Fig 5G). As controls, L685,458 reduced both the cellular generation of Aβ (Fig 5E) and γ-secretase activity (Fig 5F) while BSI IV inhibited BACE1 activity (Fig 5G). The expressions of sAPPα, APP, and secretases were not changed upon the treatments (Fig 5H). Additionally, both levodopa and piribedil stimulated Aβ generation in NSC which expresses other D_2_-like family receptors with a relatively high expression of D_4_R and a week expression of D_3_R (S2A Fig). The knockdown of D_2_R completely prevented the increase of Aβ after the treatment with levodopa or piribedil indicating that D_4_R or D_3_R may not be responsible for this effect (S2B Fig).

**Fig 6:**
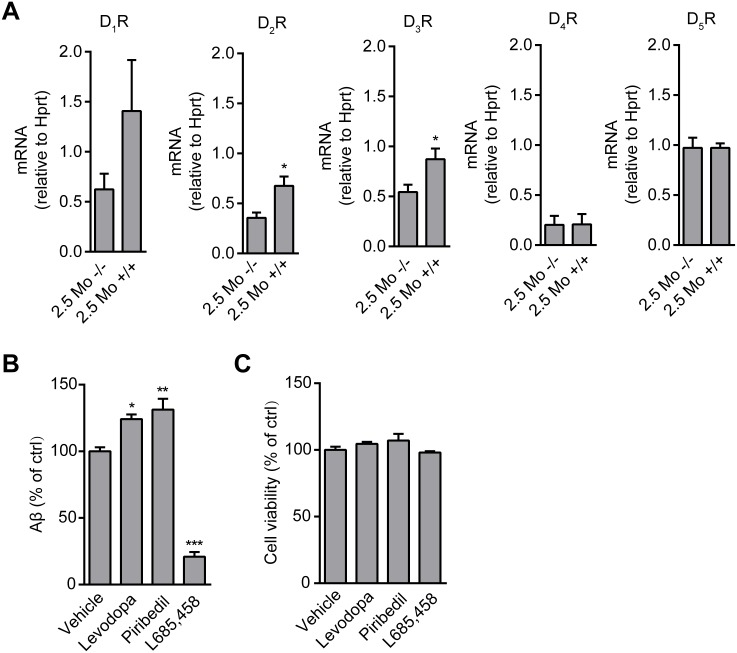
Levodopa and piribedil enhance Aβ generation in primary APP/PS1 mouse neuronal cells. (A) The mRNA level of D_1_R, D_2_R, D_3_R, D_4_R, and D_5_R in the hippocampus of WT (-/-) or APP/PS1 (+/+) mice at the age of 2.5 months (Mo). n = 4 per group. *p < 0.05 versus WT group. (B) Aβ determination after the treatment with vehicle (control), levodopa (30 nM), piribedil (30 nM) or L685,458 (1 μM) in primary APP/PS1 mouse neuronal cells. Data are mean + s.e.m., n = 6. *p < 0.05; **p < 0.01; ***p < 0.001 versus the control. (C) Cell viability measurement for the cells in (B).

We further examined the effect of levodopa and piribedil in mouse primary cultures derived from APP/PS1 mouse cortice and hippocampi. Compared to the WT mouse, the expression of D_2_R was higher in APP/PS1 mouse brain on postnatal day 0 (data not shown) and in hippocampus at the age of 2.5 month (Fig 6A). The primary hippocampal and cortical neuronal cells were isolated from postnatal day 0 APP/PS1 mouse cells and cultured for 7 days in neuronal basal medium. Levodopa and piribedil consistently stimulated Aβ generation (Fig 6B) while cell viability was unchanged indicating consistent cell number (Fig 6C).

## Discussion

Dopaminergic medications such as levodopa and dopamine receptor agonists are prescribed to improve motor deficits in PD whereas their effects on cognition are complex [7–10]. Dementias is frequent in PD with evidences indicating abnormal Aβ plaques accumulation in the brain [24–26]. Although levodopa showed protective effect in learning and memory deficits in an AD mouse model, both the Aβ plaque number and size was numerically increased [11]. Here we consistently observed increased Aβ generation after drug treatment in primary AD mouse neuron. We suspect in addition to Aβ generation, dopaminergic medications may play other functions to coordinately modulate cognition. The present study presents that levodopa and dopamine receptor agonist piribedil promote Aβ generation possibly via enhancing γ-secretase activity. Molecular mechanism study revealed the involvement of D_2_R and β-arrestin 2 implying that targeting D_2_R-mediated β-arrestin 2 pathways may have impact on Aβ pathology. We recently reported that the other class of anti-PD drugs targeting to adenosine A_2A_ receptor could also promote the generation of Aβ both in neuronal cells and in the brain of AD mouse [27]. Together with the present study, these indicate that the neurological medications targeting to certain GPCRs could have multiple effects in the CNS.

Although age-dependent reduction of D_2_R has been observed in the brain of healthy individuals [5, 6], the change of D_2_R expression with AD is debatable. An early study showed that D_2_R was reduced in the striatum of AD patients even in the absence of parkinsonian symptomatology [28] while a following evidence presented the loss of striatal D_2_R in AD with Parkinsonism but not PD or AD patients [29]. A latter microarray correlation analysis of human brain samples revealed an up-regulation of D_2_R gene expression in the patients with AD [30] and an increase of D_2_R mRNA level in the hippocampus of APP/PS1 transgenic mouse is observed in the current study. It is likely that dopamine receptors in different brain regions response differently to the disease development. Aβ is toxic to neurotransmitter pathways and can cause the impairment of dopaminergic system [31]. To compensate the reduction of dopamine release, D_2_R may up-regulated in the early stage of AD to improve dopamine function. However as the disease advances, a progressive loss of dopamine neuron results into the impairment of compensatory mechanism leading to the reduction of D_2_R and the development of disease complications such as PD. Thus, D_2_R is correlated with both PD and AD pathogenesis. Mounting evidences have shown that GPCRs modulate APP processing via multiple mechanisms. Especially for β_2_-adrenergic receptor and G protein-coupled receptor 3, β-arrestins are required. As reported, β-arrestin 2 interacts with γ-secretase complex and modulates the complex re-distribution toward detergent-resistant membranes leading to increased secretase catalytic activity [14]. In another study, β-arrestin 1 regulates the maturation of γ-secretase complex by the interaction with γ-secretase component [13]. Accordingly, it is likely that piribedil stimulates the activation of D_2_R leading to the recruitment of β-arrestin 2 to the receptor, which may further allow for the re-distribution of γ-secretase. However, β-arrestin 2 recruitment may not necessarily lead to the increased secretase activity and Aβ generation. The final outcome may also depend on receptor signaling and/or the induced subcellular distribution of β-arrestin 2/secretase complex which could be receptor or ligand specific.

Dopaminergic medications generally have multiple targets. Apart from D_2_R, levodopa could target other dopamine receptor subtypes such as D_1_R and D_3_R *in vivo* [32–34]. Piribedil could also activate D_3_R. Therefore, their effects on Aβ generation could depend on the expression pattern of these dopamine receptor subtypes in a specific cell type or brain region. Particularly, receptor expression could be altered by disease progression or drug treatment [10]. Accordingly, the cognitive effects of levodopa are influenced by the exposition duration to levodopa [35], disease stage, motor fluctuations [36], and genetic polymorphisms in levodopa metabolic pathway [37]. Another dopaminergic agent apomorphine targets both dopamine receptors and serotonin receptors with high affinity. The latter has been shown to regulate Aβ deposition [38]. We consistently found that apomorphine reduced cellular Aβ generation (data not shown). All these imply the complicated effects of dopaminergic medications on cognition *in vivo*.

## Supporting information

S1 Fig Piribedil promotes Aβ generation in HEK293/APPswe cells expressing D_2_R but not in the cells expressing D_1_R. (A) The cAMP response mediated by vehicle (control), dopamine (30 nM), or piribedil (30 nM) in HEK293 cells transfected with β-gal, D_1_R, or D_2_R. Data are mean + s.e.m., normalized to control. n = 3. ***p < 0.001 versus the control within the group. (B) The cellular Aβ level in response to vehicle or piribedil in HEK293/APPswe cells transfected with β-gal, D_1_R, or D_2_R. Data are mean + s.e.m., normalized to control. n = 3. ***p < 0.001 versus the control within the group.
(TIF)

S2 Fig Levodopa and piribedil promote Aβ generation in NSC. (A) The mRNA expressions of dopamine receptor subtypes including D_1_R, D_2_R, D_3_R, D_4_R, and D_5_R in human NSC. Data are mean + s.e.m., normalized to HPRT. n = 4. (B) The expression of endogenous D_2_R in NSC infected with scrambled or D_2_R shRNA. Actin was used as loading control. The extracellular level of Aβ in response to vehicle (control), levodopa (30 nM), piribedil (30 nM), or FSK (1 μM) in NSC infected with either scrambled or D_2_R shRNA. Data are mean s.e.m., n = 5. *p < 0.05; **p < 0.01; ***p < 0.001 versus the control within the group. FSK, forskolin.
(TIF)
