# Supplementary figures and images for: Dopamine D2 receptor and β-arrestin 2 mediate Amyloid-β elevation induced by anti-parkinson’s disease drugs, levodopa and piribedil, in neuronal cells
Source: PLoS One. 2017 Mar 2;12(3):e0173240. doi: 10.1371/journal.pone.0173240 (PMC5333886; doi:10.1371/journal.pone.0173240)

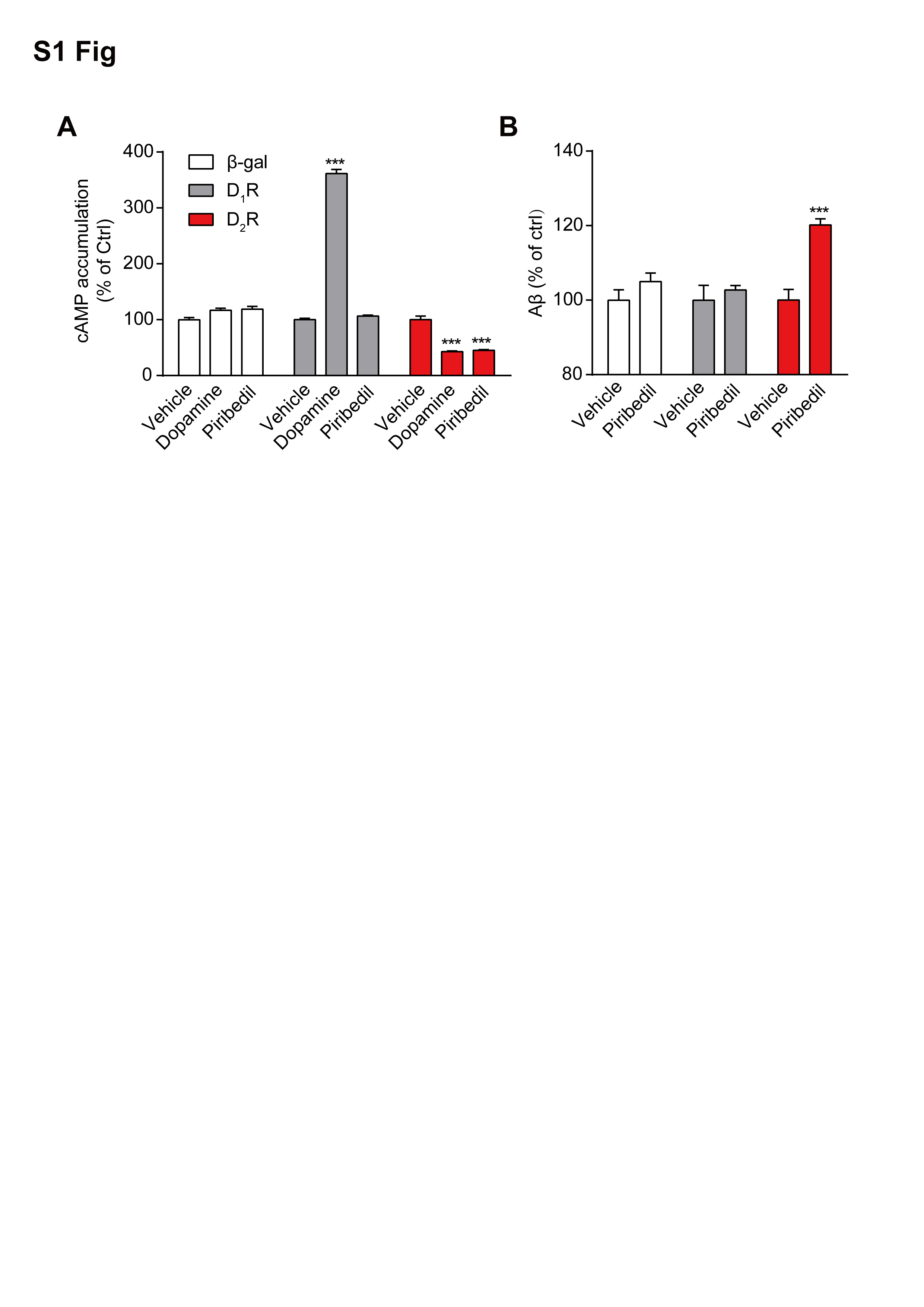

Supplement: S1 Fig — (A) The cAMP response mediated by vehicle (control), dopamine (30 nM), or piribedil (30 nM) in HEK293 cells transfected with β-gal, D1R, or D2R. Data are mean + s.e.m., normalized to control. n = 3. ***p < 0.001 versus the control within the group. (B) The cellular Aβ level in response to vehicle or piribedil in HEK293/APPswe cells transfected with β-gal, D1R, or D2R. Data are mean + s.e.m., normalized to control. n = 3. ***p < 0.001 versus the control within the group. (TIF) [file pone.0173240.s001.tif]

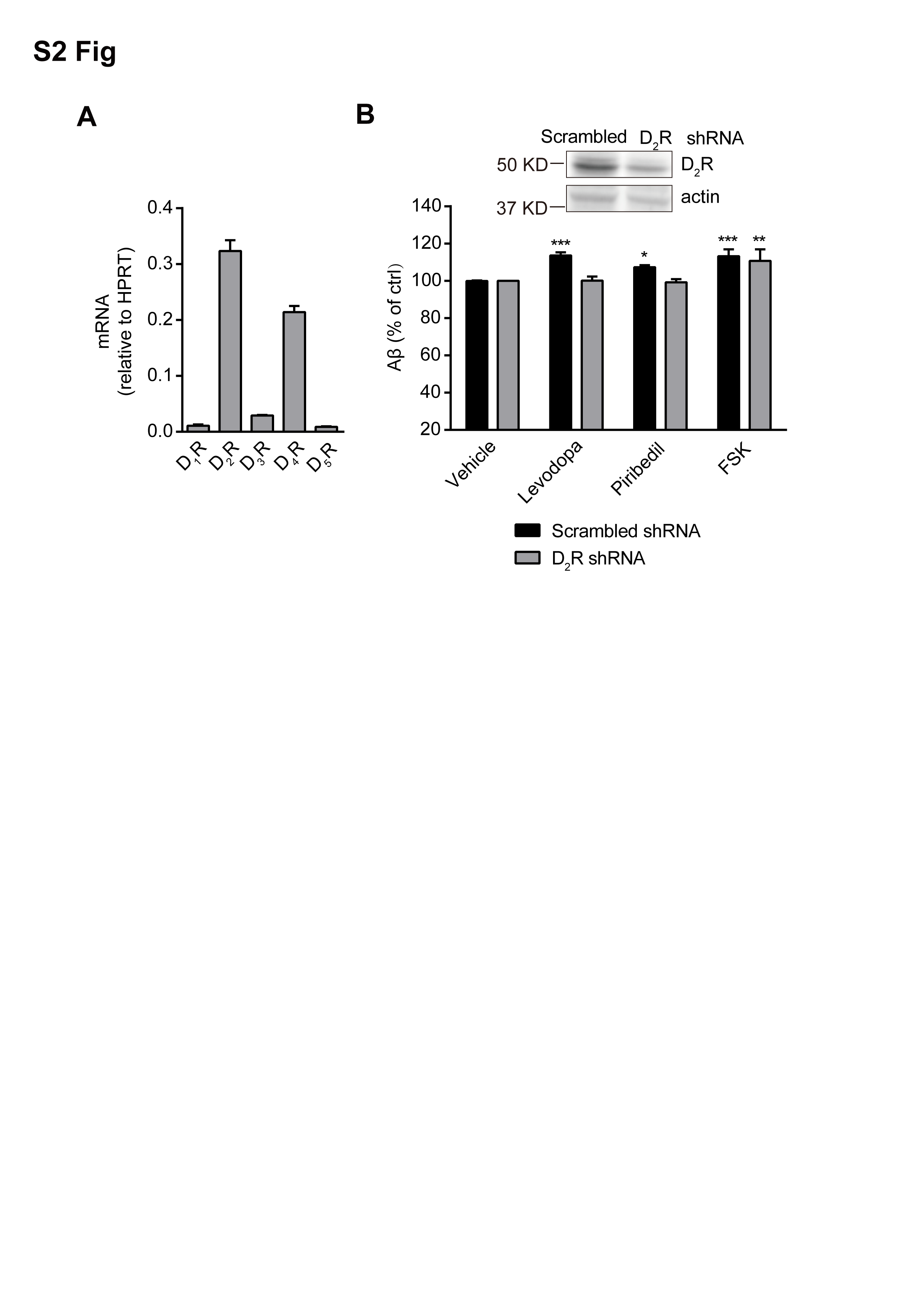

Supplement: S2 Fig — (A) The mRNA expressions of dopamine receptor subtypes including D1R, D2R, D3R, D4R, and D5R in human NSC. Data are mean + s.e.m., normalized to HPRT. n = 4. (B) The expression of endogenous D2R in NSC infected with scrambled or D2R shRNA. Actin was used as loading control. The extracellular level of Aβ in response to vehicle (control), levodopa (30 nM), piribedil (30 nM), or FSK (1 μM) in NSC infected with either scrambled or D2R shRNA. Data are mean s.e.m., n = 5. *p < 0.05; **p < 0.01; ***p < 0.001 versus the control within the group. FSK, forskolin. (TIF) [file pone.0173240.s002.tif]
